# Epidemiologic natural history and clinical management of Human Papillomavirus (HPV) Disease: *a critical and systematic review of the literature in the development of an HPV dynamic transmission model*

**DOI:** 10.1186/1471-2334-9-119

**Published:** 2009-07-29

**Authors:** Ralph P Insinga, Erik J Dasbach, Elamin H Elbasha

**Affiliations:** 1 Department of Health Economic Statistics, Merck Research Laboratories, North Wales, PA, USA

## Abstract

**Background:**

Natural history models of human papillomavirus (HPV) infection and disease have been used in a number of policy evaluations of technologies to prevent and screen for HPV disease (e.g., cervical cancer, anogenital warts), sometimes with wide variation in values for epidemiologic and clinical inputs. The objectives of this study are to: (1) Provide an updated critical and systematic review of the evidence base to support epidemiologic and clinical modeling of key HPV disease-related parameters in the context of an HPV multi-type disease transmission model which we have applied within a U.S. population context; (2) Identify areas where additional studies are particularly needed.

**Methods:**

Consistent with our and other prior HPV natural history models, the literature review was confined to cervical disease and genital warts. Between October 2005 and January 2006, data were gathered from the published English language medical literature through a search of the PubMed database and references were examined from prior HPV natural history models and review papers. Study design and data quality from individual studies were compared and analyses meeting pre-defined criteria were selected.

**Results:**

Published data meeting review eligibility criteria were most plentiful for natural history parameters relating to the progression and regression of cervical intraepithelial neoplasia (CIN) without HPV typing, and data concerning the natural history of HPV disease due to specific HPV types were often lacking. Epidemiologic evidence to support age-dependency in the risk of progression and regression of HPV disease was found to be weak, and an alternative hypothesis concerning the time-dependence of transition rates is explored. No data were found on the duration of immunity following HPV infection. In the area of clinical management, data were observed to be lacking on the proportion of clinically manifest anogenital warts that are treated and the proportion of cervical cancer cases that become symptomatic by stage.

**Conclusion:**

Knowledge of the natural history of HPV disease has been considerably enhanced over the past two decades, through the publication of an increasing number of relevant studies. However, considerable opportunity remains for advancing our understanding of HPV natural history and the quality of associated models, particularly with respect to examining HPV age- and type-specific outcomes, and acquired immunity following infection.

## Background

It is estimated that genital human papillomavirus (HPV) infection is responsible for approximately 500,000 cervical cancer cases and 275,000 associated deaths worldwide each year [1,2]. HPV has also been linked in varying degrees to cancers of the anus, vulva, vagina, penis, and head and neck, as well as anogenital warts and recurrent respiratory papillomatoses (RRP) [3-6].

In recent years, a number of natural history models of HPV disease have been developed and used in policy evaluations of the cost-effectiveness of emergent technologies to prevent and screen for HPV-related disease, such as HPV vaccination [7-10], liquid-based cervical cytology [11] and HPV testing [12,13]. Although the purpose and structure of each model have differed somewhat, a common thread across analyses has been the baseline modeling of progression and regression of HPV infection through potential outcomes of cervical cancer and death, and the overlaying of clinical diagnostic and treatment variables (e.g., potential detection of abnormal cervical cells through Pap screening) for HPV disease. Nonetheless, even where a particular parameter has been common to several natural history models, the chosen values have at times varied widely. For instance, for estimating the annual proportion of untreated cervical intraepithelial neoplasia (CIN) grade 1 lesions regressing, two economic evaluations of HPV vaccination published by Sanders et al. in 2003 [9] and Goldie et al. in 2004 [7] assumed very different parameter values and ranges (2.7–14.2% vs. ≥ 79.7%).

The degree to which variation in a parameter value will influence model output and results will depend upon the particular parameter, model, intervention and output in question. Based on the observed variation in parameter estimates in existing HPV natural history models, we elected to conduct a critical and systematic review of the literature on the epidemiologic natural history and clinical outcomes of HPV disease, in developing an HPV multi-type disease transmission model which we have applied within a U.S. population context [14] Our findings are presented here, with the goals of: (1) Providing a review and discussion of the evidence base to support epidemiologic and clinical modeling of key HPV disease-related parameters for policy evaluations; (2) Identifying areas where research data are lacking and additional studies are particularly needed.

## Methods

### Scope of review

**Figure 1:**
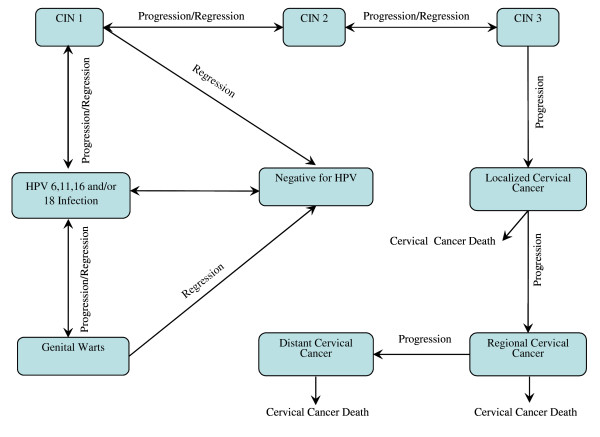
**Overview of epidemiologic structure of multi-HPV type model**. HPV infection may progress to either genital warts or cervical disease, with regression possible for HPV infection, CIN grades 1–3 and genital warts. Only cervical cancer confers an added risk of mortality, as depicted in the figure. However, in the full model (not shown for simplicity) all individuals face an underlying age and sex-specific mortality rate due to non-cervical cancer-related causes. CIN = Cervical Intraepithelial Neoplasia; HPV = Human Papillomavirus.

**Figure 2:**
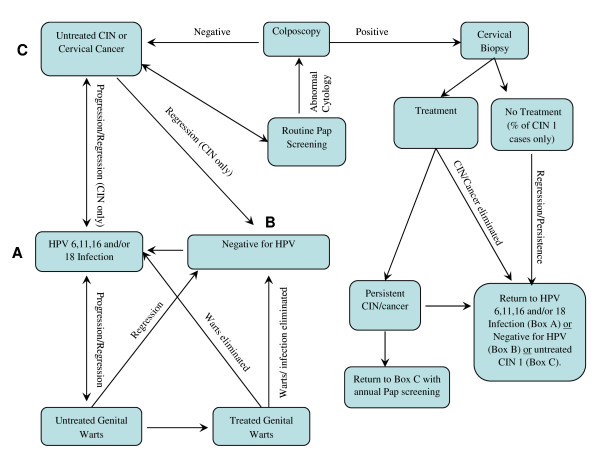
**Overview of clinical structure of multi-HPV typemodel**. HPV infection may progress to either genital warts or cervical disease, with regression possible for HPV infection, CIN grades 1–3 and genital warts. Treated genital warts, CIN and cervical cancer may result in disease eradication with elimination of HPV infection, disease eradication with persistent HPV infection, or failure to eradicate disease or HPV infection. Once CIN is detected, women are followed with annual Pap screening. Women in all health states are also subject to an age-specific rate of hysterectomy for benign conditions (not shown for simplicity). CIN = Cervical Intraepithelial Neoplasia; HPV = Human Papillomavirus.

Overviews of the epidemiologic and clinical structure of our HPV multi-type model are presented in Figures 1 and 2. In the model, incident HPV 6, 11, 16 and 18 infections arise through sexual mixing of males and females in the population. The sexual mixing matrix, along with a description of other model components (e.g., population demographic characteristics, economic costs of care, health-related quality of life) are described elsewhere [14]. The present systematic review focuses upon natural history model health state transitions ranging from HPV infection through HPV disease and associated mortality. Unlike sexual mixing, these parameters are generally common to most prior cohort-based and dynamic transmission models of HPV disease [10-13].

Consistent with the model, our review is also confined to the epidemiologic natural history and clinical outcomes of cervical HPV disease and genital warts. Prior HPV natural history models to ours had generally examined only a single facet of HPV disease [7-9,15] and the incorporation of both cervical disease and genital warts into a single model represented a step forward. However, further research is on-going to evaluate and incorporate the natural history of other HPV-related diseases (e.g., other genital cancers, head and neck cancer and RRP). Also, in the model and our present review, we focus specifically upon the natural histories of HPV 6, 11, 16 and 18 infections as HPV types 16 and 18 are responsible for approximately 70% of cervical cancers [16] and types 6 and 11 the vast majority of genital warts[17,18] and reflect the types targeted by one or both of the prophylactic HPV vaccines [19,20], which were the subject of our model technology assessment [14]. Few data are currently published on disease natural history relating to other HPV types, but we may expand our model to include additional HPV types as such information becomes available.

Certain clinical parameters (i.e., rates of Pap screening/coverage, rates of hysterectomy, cervical cancer mortality by stage) would be expected to vary by country. For instance, Pap testing coverage and hysterectomy rates may vary according to local guidelines, resources and cultural factors and cervical cancer mortality rates by stage in relation to the availability of specific treatments. In this review, parameters most likely to vary by country are described for the U.S. population, however data source selection issues may also be applicable to other settings. Where applicable, for further illustration, we additionally reference data sources used in adaptations of our model to selected other country settings. Finally, although often sharing many parameters in common, it is recognized that heterogeneity exists in the structure of different HPV natural history models. For instance, some models lump CIN 2 and CIN 3 into a single health state (CIN 2/3) [13,21,22], while others model them as separate states [14,23,24]. A discussion of data to inform parameters for all possible permutations in model structure is beyond the scope of this review, however, data sources and methodologic issues discussed for the particular model structure illustrated in the present study (Figures 1 and 2) will in large part have applicability to other models as well.

### Literature Review Design

For development of our model, between October 2005 and January 2006, data were gathered from the published English language medical literature through searches of the PubMed database as well as from published reports and statistics available on the internet and CD-ROM. References were also examined from prior HPV natural history models [8,9,11,13,21-26] and review papers [27-30]. Because the parameters encompassed within the model were diverse, ranging from HPV infection to cervical cancer to hysterectomy, separate literature searches within PubMed were conducted for ten different parameter groups: (1) Duration and progression of cervical HPV infection and natural immunity; (2) Progression and regression of cervical intraepithelial neoplasia; (3) Natural history of cervical cancer; (4) Hysterectomy; (5) Cervical cytology screening; (6) Cervical cytology sensitivity and specificity; (7) Symptom development for cervical cancer; (8) Eradication of HPV disease with treatment; (9) HPV persistence following treatment; (10) Anogenital wart patients seeking physican care. The specific constellations of search terms utilized for each of these parameter groups, along with a listing of the number of articles retrieved from PubMed and selected for this review, are reported in detail in Table 1.

**Table 1:** Description of Literature Search

Category	Search terms	Articles Retrieved	Articles Selected for this Review
Duration and progression of cervical HPV infection and natural immunity	<human papillomavirus and cervical> OR <human papillomavirus and cervix>	7,454	8

Progression and regression of cervical intraepithelial neoplasia	<cervical intraepithelial neoplasia and progression> OR <CIN and progression> OR <cervical intraepithelial neoplasia and natural history> OR <CIN and natural history> OR <cervical intraepithelial neoplasia and regression> OR <CIN and regression> OR <cervical intraepithelial neoplasia and clearance> OR <CIN and clearance>	1,321	5

Natural history of cervical cancer	<cervical and cancer and natural history> OR <cervix and cancer and natural history> OR <cervical and cancer and progression> OR <cervix and cancer and progression>	2,538	4

Hysterectomy	<hysterectomy and rate and United States>	270	1

Cervical cytology screening	<Pap and rate and United States> OR <cervical and screening and rate and United States> OR <cervix and screening and rate and United States> OR <cervical and cytology and rate and United States> OR <cervix and cytology and rate and United States>	848	3

Cervical cytology sensitivity and specificity	For literature published up to October 1999 we consulted a prior systematic review:	559	2
	Nanda K, McCrory DC, Myers ER, Bastian LA, Hasselblad V, Hickey JD, Matchar DB. Accuracy of the Papanicolaou test in screening for and follow-up of cervical cytologic abnormalities: a systematic review. Ann Intern Med. 2000 May 16;132(10):810–9.		
	For literature published from October 1999 forward we used search terms of:		
	<Pap and cervical intraepithelial neoplasia and sensitivity> OR <cytology and cervical intraepithelial neoplasia and sensitivity> OR <screening and cervical intraepithelial neoplasia and sensitivity> OR <Pap and cervical intraepithelial neoplasia and specificity> OR <cytology and cervical intraepithelial neoplasia and specificity> OR <screening and cervical intraepithelial neoplasia and specificity> OR <Pap and CIN and sensitivity> OR <cytology and CIN and sensitivity> OR <screening and CIN and sensitivity> OR <Pap and CIN and specificity> OR <cytology and CIN and specificity> OR <screening and CIN and specificity>		

Symptom development for cervical cancer	<cervical and cancer and symptom> OR <cervix and cancer and symptom>	255	0

Eradication of disease with treatment	<cervical intraepithelial neoplasia and treatment and loop excision and recurrence> OR <cervical intraepithelial neoplasia and treatment and LEEP and recurrence> OR <cervical intraepithelial neoplasia and treatment and LLETZ and recurrence> OR <cervical intraepithelial neoplasia and treatment and loop excision and residual> OR <cervical intraepithelial neoplasia and treatment and LEEP and residual> OR <cervical intraepithelial neoplasia and treatment and LLETZ and residual> OR <cervical intraepithelial neoplasia and treatment and loop excision and failure> OR <cervical intraepithelial neoplasia and treatment and LEEP and failure> OR <cervical intraepithelial neoplasia and treatment and LLETZ and failure> OR <genital wart and treatment and recurrence>	362	10

HPV persistence following treatment	<human papillomavirus and treatment and persistence> OR <human papillomavirus and treatment and clearance>	162	2

Anogenital wart patients seeking physican care	<genital wart and untreated> OR <genital wart and undiagnosed> OR <genital wart and care seeking>	36	0

**Name of host: **PubMed http://www.ncbi.nlm.nih.gov/sites/entrez

**Dates of search: **October 2005–January 2006

**Years covered by search: **1950–2006

**Language: **English language literature

**Complete search strategy used: **Different search terms were used for various components of HPV infection and disease epidemiology and clinical management. These terms are summarized here by category.

### General Methodologic Criteria

A number of prior analyses have discussed methodological challenges in describing the natural history of HPV disease [27,29-31], and several explicit choices concerning alternate data sources and study designs were made in the present review. Given the diversity of model parameters examined, data sources and selection criteria unique to specific model parameters will be discussed within their respective sections in the Results, with an overall summary provided in Table 2. This section describes general literature review methodologic criteria applicable to multiple parameters.

**Table 2:** Summary of Study Selection Criteria By Parameter Group

Parameter Group	Study Selection Criteria
*General criteria for stud y selection*	• Nationally representative studies meeting selection criteria
	➢ If unavailable, then select broad population-based studies
	▪ If unavailable, then select local studies
	• Specificity of results to HPV type groupings of interest (16/18 or 6/11)
	➢ If studies specific to HPV 16/18 or 6/11 infection or disease are unavailable, then select studies of all high-risk or all low-risk HPV types, respectively
	▪ If unavailable then select studies for all infections or disease
	• PCR-based methods for HPV detection in infections

Progression of HPV infection and disease	• Histologic confirmation of cervical disease
	• Data available for outcomes reported over a 12-month time horizon

HPV infection mean duration in absence of detectable disease	• Specificity of results to HPV type groupings of interest (16/18 or 6/11)
	• Truncation of infection duration at time of disease detection via histology
	• Limited degree of censoring beyond longest infection follow-up time

Regression of HPV infection and disease	• Histologic confirmation of cervical disease at baseline
	• Biopsy confirmation of cervical HPV-type specific disease absence during follow-up to connote regression
	➢ If unavailable for all cases, then select studies with either biopsy confirmed HPV-type specific disease absence for a portion of cases, with negative cytology for non-biopsied cases, OR biopsy confirmed disease absence, irrespective of HPV-type
	• Data available for outcomes reported over a 12-month time horizon

Cervical cancer mortality	• Data available on an age- and stage-specific basis
	• Nationally representative or broad population-based studies in unscreened women
	➢ If unavailable, then select nationally representative or broad population-based studies in screened and unscreened women
	• Data available for outcomes reported over a 12-month time horizon

Hysterectomy for non-HPV related conditions	• Age-specific annual hysterectomy rates reported

Cytology screening rates	• Age-specific annual routine cervical cytology screening rates reported
	➢ Routine screening reported separately from follow-up screening
	➢ Cervical cytology reported separately from vaginal cytology
	• Data based on documented screening utilization in a population-based study if available
	➢ If unavailable, then select studies based on patient self-report

Cytology sensitivity	• Liquid-based cytology evaluated
	• Cervical biopsy performed on all women
	➢ If unavailable, then select studies in which cervical biopsy was performed on at least a random sample of women with negative cytology and colposcopy results

Cytology specificity	• Liquid-based cytology evaluated
	• Cervical colposcopy performed on all women, with biopsy performed if abnormalities suspected
	• Biopsy results reported for all grades of cervical disease (≥ CIN 1)

Colposcopy sensitivity/specificity	• Colposcopy performed following abnormal cytology
	• Colposcopically directed cervical biopsy performed on all women
	• Biopsy results reported for all grades of cervical disease (≥ CIN 1)

Symptom development among cancer patients	• Stage-specific symptom development
	• Representative cross-section of patients with cervical cancer at each stage including patients who may harbor occult cancers
	➢ If unavailable, then rely upon expert opinion from the literature

Eradication of CIN with treatment	• Representative study of CIN therapies used in practice if available
	➢ If unavailable then select studies of LEEP (most common modality)
	• Stratified reporting of outcomes by pre-treatment CIN grade
	• Post-treatment follow-up of all women within 12 months via colposcopy and/or biopsy
	• Definition of recurrent or residual disease as CIN 1 or more severe histology

Eradication of cervical cancer with treatment	• Nationally representative or broad population-based studies of 5-year disease-free survival by cancer stage
	➢ If unavailable, then select nationally representative or broad population-based studies of 5-year relative survival by cancer stage

Eradication of genital warts with treatment	• Representative study of genital wart treatments used in clinical practice
	• Physician ascertained clearance following treatment for all subjects

Persistence of HPV following cervical disease eradication	• Representative study of therapies used in practice if available
	➢ If unavailable, then select studies of LEEP (most common modality) for CIN, and hysterectomy or radiation therapy for cervical cancer
	• Histologic confirmation of disease pre-treatment and post-treatment (for exclusionary study purposes)
	• HPV typing of pre- or post-treatment lesion tissue specimens or both
	➢ If unavailable, then select studies with HPV typing of any cervical specimen
	• Follow-up for all women within 6 months post-treatment
	➢ If unavailable, then select studies with less prompt follow-up
	• Colposcopy performed on all women post-treatment to assist in confirming disease eradication

Persistence of HPV following genital wart eradication	• Representative study of genital wart treatments used in clinical practice
	• Testing for HPV infection across a range of anogenital sites post-treatment (not just at the former wart site)
	• Follow-up for all women within 6 months post-treatment
	➢ If unavailable, then select studies with less prompt follow-up

Care seeking behavior for genital warts	• Population-based studies of patients with genital warts, including both those who have, and who have not, chosen to seek physician care
	➢ If unavailable, then rely upon expert opinion from the literature

CIN = Cervical intraepithelial neoplasia; HPV = Human papillomavirus; LEEP = loop electrosurgical excision procedure; PCR = Polymerase chain reaction

#### Cytology vs. Histology

The multi-HPV type mode; [14] (henceforth, the model), like others previously developed, is structured to allow for progression and regression between cervical histological, rather than cytological, health states. Therefore, in assessing the natural history of cervical intraepithelial neoplasia (CIN) and cancer, only studies with histologically confirmed disease were eligible for inclusion as data sources. While less invasive, studies that use cervical cytologic diagnoses (e.g., atypical squamous cells of undetermined significance [ASCUS], atypical glandular cells [AGC], low-grade squamous intraepithelial lesion [LSIL], high-grade SIL [HSIL]), rather than histology (CIN 1–3), are viewed as less reliable proxies for the presence and grade of underlying CIN. For instance, in the ASCUS/LSIL Triage Study (ALTS), biopsying only women with cytologic diagnoses of = HSIL was observed to lead to ~60% fewer diagnoses of CIN 2/3 than performing colposcopy and biopsy on all women, or triaging women to colposcopy based on a high-risk positive HPV test. Also there was a substantially smaller proportion of women with HSIL Pap smears (30–35%) than were diagnosed with CIN 2/3 in the other trial arms [32]. In addition to the potential for missing more true disease, there is also the potential for either disease misclassification, or a false positive result on cytology. Even for HSIL cytology, in the ALTS trial, 40% of cases were followed by a histological diagnosis less severe than CIN 2/3 during follow-up, without further treatment [32].

It has been hypothesized that biopsies may alter the natural history of cervical disease [30,31], which has at times been used to support the use of cytologic versus histologic natural history study data. A randomized clinical trial using digital imaging colposcopy of women undergoing no biopsy, central biopsy and peripheral biopsy at baseline found no significant difference in change in lesion size across the three groups at 6 week follow-up [33]. Furthermore, loop electro-surgical excision procedures (LEEPs) performed at the same visit confirmed that none of the CIN 1–3 cases detected on punch or peripheral biopsy were eliminated due to the biopsy procedure. Recent reviews of the issue have concluded that while the use of larger wedge biopsies in early studies may have led to greater concerns regarding alteration of CIN natural history, there is not evidence to suggest that the small volume of tissue removed in central biopsies significantly affects the natural course of disease [31,34].

#### Classification and Quality of HPV Testing and Typing

The past two decades have seen major advances in the quality of HPV testing and typing techniques available. Early studies characterized the presence or absence of HPV infection using Pap smear cytologic impressions [35], dot filter hybridization [36], southern blot hybridization [37] and other techniques. In studies involving the detection of individual HPV types, polymerase chain reaction (PCR) techniques are now widely used over these other methods and have generally been shown to be of higher quality [38,39]. In describing the type-specific natural histories of HPV 6, 11, 16 and 18 infections, studies utilizing non-PCR-based methods for the detection of HPV DNA were therefore excluded from the review.

Also, the model separately simulates the natural histories of HPV 6/11 and HPV 16/18 infections and focuses solely on disease due to these HPV types. Thus, in deriving model inputs, studies that provided data on the natural history of HPV 16/18 disease were preferred over those describing data for disease due to all high-risk (H-R) HPV types, which in turn were preferred over those with data for disease due to all HPV types (H-R and low-risk [L-R] combined). A similar hierarchy was employed for selecting studies in the modeling of HPV 6/11 infection.

The combining of data into two groupings for types 6/11, and 16/18, respectively, was performed due to a general lack of published studies specifically describing the time to progression of individual incident HPV types in the development of disease at the time of our initial model design. The few available studies with stratification by HPV type combined reporting of results for HPV types 6/11 and 16/18 [36,40,41]. In our own work, we have begun to describe the natural history of individual HPV types and have observed similar infection durations for HPV 6 and 11, and HPV 16 and 18, respectively [42]. With regard to disease progression, confidence intervals for individual type progression rates have either been similar and overlapping, or sample sizes too small regardless (in the case of HPV 11) for meaningful comparisons between paired types [43]. As additional data on single type infections are published, further refinements may become possible. Given that other HPV natural history models may have alternate structures as compared to that of our model [10,44], studies presenting data with other HPV type dichotomies will also be briefly discussed.

#### Interval of Follow-up

The model uses continuous mathematical functions to estimate the instantaneous rates of progression and regression of disease. In practice, however, all studies of HPV disease natural history collect observations at discrete time intervals, typically ranging from several months to several years apart. Given the relatively active progression and regression of some facets of HPV disease within a matter of months [45,46], differences in follow-up intervals across studies or patients within a given study can produce vastly different results regardless of differences in underlying natural history [34].

For instance, suppose a woman developed an incident CIN 1 lesion just prior to baseline, progressed to CIN 2 at 12 months and regressed to negative for HPV infection at 24 months. A study with observations at only baseline and month 24 would conclude that this CIN 1 case regressed to normal, whereas a two-year study with a 12 month observation interval would observe progression to CIN 2, as well as subsequent regression. Given that many U.S. women undergo routine Pap screening as frequently as once a year [47], with the potential for disease detection, natural history studies with observation intervals less frequent than every 12 months were excluded. Also excluded were studies that did not characterize the timing of progression and regression within uniform intervals across all women.

A decision was also made to pre-specify a uniform criterion across studies for the time point at which to assess event rates. Some HPV natural history studies report the regression or progression of CIN over a single year of total follow-up [46,48]. while others track events across multiple years [41,49]. Although studies with longer follow-up might be expected to be more comprehensive in describing disease natural history, we chose to model annual transition probabilities based on event rates observed during the first 12 months of follow-up in each study for our base case parameter estimates.

There were two main reasons for choosing a 12 month horizon. First, for a number of natural history studies, HPV typing data for lesions observed on follow-up are unavailable, and as one looks over longer time horizons, it may become increasingly likely that apparent "progression" to higher grade HPV disease, or persistence, will be unrelated to the original HPV disease state of interest. For instance, a recent study found that among 16–27 year old U.S. women with incident HPV 6, 11, 16 and 18 infections who were observed to develop CIN during up to 36 months of follow-up, 50.4% had a lesion containing a different HPV type than that observed in the incident infection [43]. While it is not possible to completely eliminate this source of bias in these types of HPV natural history studies, it was felt that data on disease activity observed during the first 12 months of follow-up would provide a more reliable proxy for annual transition rates between states. Second, estimated annualized risks of progression/regression can become meaningless if computed at or beyond the time point at which all potential transitions occur. For instance, all incident HPV 6/11 infections were observed to either progress or regress within 18 months in a recent study [43]. Because a number of natural history studies provide only a partial picture of overall transitions (e.g., disease progression only), the uniform 12 month interval was chosen for the primary analysis.

Where studies of HPV disease natural history with multiple years of follow-up are available, we will also compare the 12 month results across studies used in the base case to those observed through the mean or median follow-up time (if the mean is not reported). The choice of mean/median follow-up time is based on the observation that differential patient follow-up, as well as loss to follow-up, can render estimates derived from the longest follow-up times unstable. Unless otherwise noted, consistent with prior studies [10,12,44], a simplifying assumption for both estimations is that risks of progression and regression are generally constant over time.

#### Age-specific Natural History Modeling

We considered the issue of whether to model HPV infection and disease natural history on an age-specific basis, or uniformly across age. Recent studies have failed to demonstrate a difference with age in the rate of clearance of HPV infection [50,51], however, less data are available concerning the age-specific progression and regression of CIN. A retrospective study by Konno et al. reported that 48% of untreated CIN 2/3 cases among women age 30–49 (n = 128) were observed to progress over time compared to 32% of cases among women age 50–79 (n = 66), however, the difference in rates with age was not statistically significant [52]. In the absence of definitive data demonstrating age-specific variation in the progression and regression of CIN (or HPV), we therefore elected to apply constant risks across age.

#### Risks vs. Rates

Nearly all HPV natural history studies report a risk of progression or regression of disease, rather than a rate. Most models, however, use rates to express health state transitions. For ease of reference with respect to the original sources, we describe data selected from the literature in the form of a risk (proportion) in this review, and for our cost-effectiveness model [14]. These risks may be converted to rates using the formula *rate *= -[ln(1-*p*)]/*t*, where *p *is the risk or proportion and *t *is the follow-up time in years.

## Results

### Epidemiologic Natural History

Table 3 summarizes the data sources, parameters and values for the HPV epidemiologic natural history model variables which will now be reviewed.

**Table 3:** Epidemiologic Natural History Model Parameters

Parameter	Estimate
Progression in the presence of HPV 16/18, % per year	
Normal to CIN1[43]	9.4
Normal to CIN 1 to CIN 2 [41,43]	5.8
Normal to CIN 1 to CIN 2 to CIN 3 [41,43]	3.5
CIN1 to CIN 2*	13.6
CIN 2 to CIN3 (severe dysplasia) [49,65]	14.0
CIN 3 (severe dysplasia) to CIN 3 (CIS) [49,68]	43.0
CIN 3 (CIS) to LCC	4.1
LCC to RCC [7,9,22,23]	10.0
RCC to DCC [22]	30.0
Progression in the presence of HPV 6/11, % per year	
Normal to CIN1 [43]	8.5
Normal to CIN 1 to CIN 2 [43]	1.9
Normal to CIN 1 to CIN 2 to CIN 3 [43,78,79]	0.0
CIN 1 to CIN2 *	0.0
Normal to genital warts [41]	57
Mean HPV infection duration with CIN absent, years	
HPV 16/18 infection [42]	1.2
HPV 6/11 infection [42]	0.7
Duration of acquired immunity following HPV infection	10 years to Lifelong
Regression of HPV 16/18+ disease, % per year	
CIN1 to Negative/HPV 16/18 [40]*	32.9
CIN 2 to Negative/HPV 16/18 [49,65,85]	21.0
CIN 2 to CIN 1 [65]	13.3
CIN 3 (severe dysplasia) to Negative/HPV 16/18 [49]	11.0
CIN 3 (severe dysplasia) to CIN 1 [49,65]	3.0
CIN 3 (severe dysplasia) to CIN 2 [49,65]	3.0
Regression of HPV 6/11+ disease, % per year	
CIN1 to Negative/HPV 6/11*	55.2
Genital warts to Negative/HPV 6/11 [41,88]	87.5
Age and stage-specific cervical cancer mortality, 1997–2002, % per year [69]	
for LCC	
15–29 years	0.7
30–39 years	0.6
40–49 years	0.8
50–59 years	1.9
60–69 years	4.2
≥ 70 years	11.6
for RCC	
15–29 years	13.4
30–39 years	8.9
40–49 years	11.0
50–59 years	10.1
60–69 years	17.6
≥ 70 years	28.6
for DCC	
15–29 years	42.9
30–39 years	41.0
40–49 years	46.7
50–59 years	52.7
60–69 years	54.6
≥ 70 years	70.3

CIN = cervical intraepithelial neoplasia; CIS = carcinoma in situ; DCC = distant cervical cancer; HPV = human papillomavirus; LCC = localized cervical cancer; RCC = regional cervical cancer

*R. Insinga, unpublished data.

#### Progression of HPV 16/18 infection to Histologically Detectable CIN

##### HPV 16/18 infection to CIN 1

Only one prior study was found estimating the incidence of CIN 1 following HPV 16/18 infection [43]. The 12 month risk of progression of incident HPV 16/18 infections (n = 204) to histologically detectable CIN 1 was estimated to be 9.4% in a study by Insinga et al., one of the authors of this review. In that study, women underwent cervical swab PCR and thin-layer Pap testing at approximate 6 month intervals through 48 months of follow-up. The study protocol mandated that women with cytologic evidence of HSIL or repeated tests showing LSIL, ASCUS or AGC undergo colposcopy. However, some women were referred for colposcopy more frequently than required based on local standards of care. All women attending the month 48 trial visit were referred for colposcopy, with cervical biopsies performed if a lesion was suspected. Cervical biopsy specimens were typed for individual HPV types by PCR. If analyzed through the study mean follow-up time of 21.9 months, the cumulative risk of progression to CIN 1 was 14.6%, approximately equivalent to an annual progression rate of 8.3%. For the purposes of this review, a strength of the study was the focus specifically on HPV 16/18 infections and the correlation between HPV types observed in infection and CIN lesions. However, a limitation was that not all women in the study underwent cervical biopsy, with the potential for missed lesions.

Although no additional studies of CIN 1 incidence were found in the literature specific to HPV 16/18 infection, Hoyer et al. [53,54], followed up women with a positive H-R HPV test at baseline with colposcopy and biopsy within 8 months and reported CIN 1 cases detected. However, it was not possible to determine whether prevalent CIN disease was already present at baseline and if the HPV type observed at baseline was the same as that of the CIN lesions detected during follow-up.

##### HPV 16/18 infection to CIN 2/3

While some women with incident HPV 16/18 infections will be found on subsequent histology to have CIN 1 in natural history studies, there are also instances where the transition from HPV to CIN 1 to CIN 2 may occur before an initial histologic specimen is taken, and a CIN 1 diagnosis is never observed [36,55,56]. In some cases, there will even be rapid enough progression from incident HPV infection to CIN 1 to CIN 2 to CIN 3 to render CIN 3 the first detectable CIN state for the purposes of modeling.

The 12-month risk of progression of incident HPV 16/18 infection to CIN 2 was estimated to be 5.8%, and to CIN 3 was estimated to be 3.5%, based on studies by Winer et al. and Insinga et al. [41,43]. In the Winer et al. study, women underwent Pap screening, colposcopic evaluation and cervical swab HPV testing and typing at 4 month intervals. Women with cytologic or colposcopic evidence of HSIL, or repeated LSIL or equivocal cytology, were biopsied. The combined incidence of CIN 2–3 within 12 months following an incident HPV 16/18 infection (n = 60) was observed to be ~12%. Five cases of CIN 2 and four cases of CIN 3 had HPV 16/18 infection detected on a cervical swab at the visit prior to biopsy, with no difference observed in the median time from incident HPV infection to incident CIN 2 vs. CIN 3. Annual risks of progression to CIN 2 (12%*5/9 = 6.7%) and CIN 3 (12%*4/9 = 5.3%) were derived from these figures. In the Insinga et al. study, described previously, the 12 month risk of progression to CIN 2 was 4.8% and to CIN 3 was 1.7% [43], which were averaged with the Winer et al. data to yield overall risks of progression.

The Winer and Insinga studies also included data on incident HPV 16/18 infections through a mean follow-up of 38.8 and 21.9 months respectively, with respective cumulative risks of progression to CIN 2/3 during those time intervals of approximately 27.2% and 11.5%, or 9.4% and 6.5%, when annualized [41].

The Winer study has a number of strengths for assessing HPV natural history with respect to CIN 2/3 development relative to other analyses [36,53], including the identification of type-specific incident HPV infections, frequency of follow-up, and PCR testing used for HPV detection and typing. A few limitations of the study, in the context of the present review, were that all CIN 2–3 biopsy results, regardless of underlying HPV type in the lesion, were used in estimating incidence rates of CIN 2–3 following HPV 16/18 infection, some women with an impression of LSIL on colposcopy or who appeared normal who were never biopsied may have harbored underlying CIN 2–3, and some women may have had CIN 1 detected prior to their CIN 2/3 diagnosis [41]. The Insinga study overcame the first and last of these limitations, and the colposcopic threshold for biopsy was lower (LSIL vs. HSIL), however, colposcopy was less routinely performed than in the Winer study. Other published data overcoming these limitations were not found.

#### Progression of HPV 16/18-positive CIN and Cancer

##### HPV 16/18-positive CIN 1 to CIN 2

No studies were found describing the 12 month progression of HPV 16/18-positive CIN 1 to CIN 2. Three studies reported data on the 12 month progression of biopsy-confirmed CIN 1 to CIN 2 without regard to HPV type [48,49]. Twelve month risks of progression across the three studies averaged ~7%. However, based on a review of U.S. studies reporting HPV type-specific PCR testing results for CIN 1 biopsy specimens, approximately 15% of all CIN 1 cases are estimated to be associated with HPV 16/18 infection, with the remainder associated with other HPV types or testing negative for HPV (~18%) [43,58-61]. Furthermore, a similar review of U.S. studies of biopsy-confirmed CIN 2–3 found that 59% of specimens tested positive for HPV 16/18 infection [58-62], suggesting that HPV 16/18 positive CIN 1 is more likely to progress to CIN 2 than CIN 1 due to other HPV types. As such, studies reporting data across all CIN 1 cases were deemed to be of low adequacy for assessing the natural history of CIN 1 due to HPV 16/18 infection.

Since type-specific data were not found in the literature, we instead examined data from Merck's F.U.T.U.R.E. I and II HPV vaccine phase III trials [63]. Data were analyzed for placebo arm women, age 16–23 at baseline, who developed incident HPV 16 or 18 infection during the course of follow-up as detected through cervical swabs, and who subsequently were detected with incident biopsy-confirmed CIN 1 that tested positive on Thinsection PCR testing for HPV 16 or 18 (R. Insinga, unpublished data). Women with incident infection for a specific HPV type were required to be sero-negative and PCR negative for that type at baseline, and to qualify as an incident CIN 1 cases due to a particular HPV type there must have been no biopsy result with higher grade CIN or cancer testing positive for that HPV type prior to the CIN 1 diagnosis. Once CIN 1 was diagnosed, women were followed up with repeat cytology at 6 month intervals, with repeat colposcopy and biopsy if cytologic abnormalities persisted. Four women with persistent CIN 1 on 2–4 consecutive biopsies were treated during the course of follow-up, and evaluated as censored at the time of treatment. Among 64 women with incident CIN 1 biopsy specimens testing positive for HPV 16/18 infection, the 12 month risk of progression to HPV 16/18 positive CIN 2 was estimated to be 13.6%. None of the women progressed directly from CIN 1 to CIN 3. There were insufficient data beyond 12 months follow-up for longer-term analyses.

A limitation of the Merck trial-based analysis is that generally only women with continued cytologic abnormalities during the course of follow-up underwent repeat colposcopy and biopsy. Also, if the small number of women with persistent CIN 1 who were treated differed in their natural history from those with persistent CIN 1 who were untreated at the same follow-up time, the results could be affected. The finding that the 12-month risk of progression of CIN 1 due to HPV 16/18 (13.6%) was higher than that averaged from analyses reporting data for all CIN 1 cases (~7%) is consistent with the greater known risk of high-grade CIN and cancer due to these types [41,64].

##### HPV 16/18-positive CIN 2 to CIN 3 (severe dysplasia)

No studies were found in the literature review documenting the 12-month progression of CIN 2 due to HPV 16/18 infection to CIN 3 or for any higher grade of cervical disease due to HPV 16/18 infection. For U.S. women in the placebo arms of F.U.T.U.R.E. I and II, it was estimated that 54% of initial CIN 2 biopsies (n = 37), that were not preceded by a higher grade of CIN or cancer, tested positive for HPV 16/18 infection. Thus, studies of disease regardless of HPV type status are likely to be more generalizable to HPV 16/18 disease natural history for CIN 2 than CIN 1.

Two studies were identified reporting a 12 month risk of progression of biopsy confirmed CIN 2 to CIN 3 (severe dysplasia). In the first, by Kataja et al. (n = 70), women with CIN 2 on punch biopsy at baseline were followed up with Pap screening, colposcopy and biopsy at 6 month intervals, with treatment of disease if progression to carcinoma in situ (CIS) was observed on biopsy [49]. The second, by De Aloysio et al., was a clinical trial of interferon-β treatment of CIN 2 [65]. Only data for placebo arm participants (n = 15) were included in the present review, as these women were not treated unless progression to CIN 3 was observed on biopsy. Similar to the Kataja study, women underwent colposcopy and biopsy at 6 month intervals during follow-up. Progression in both studies was defined based on a biopsy confirmed diagnosis of CIN 3, with ~8% of women in the Kataja study and 20% of women in the De Aloysio study progressing to CIN 3 within 12 months. Averaging across the two studies yielded an annual risk of progression of 14%.

The mean follow-up time in the Kataja study was 3.75 years and in the De Aloysio study was 2.0 years. Progression to CIN 3 through the mean follow-up time in the Kataja study was ~23% and in the De Aloysio study was 33%. The corresponding estimated annual proportion progressing through the mean follow-up times were 7% and 18% respectively (mean 12%), similar to the estimates derived from the 12 month follow-up data. A limitation of both analyses in the context of the present review is that they did not report data specifically for HPV 16/18 disease and hence did not verify that CIN 3 cases observed over time were due to the same HPV types found in the CIN 2 lesions.

##### HPV 16/18-positive CIN 3 (severe dysplasia) to CIN 3 (CIS)

The histologic diagnosis of CIN 3 may be sub-divided into the categories of severe dysplasia, and CIS, representing conversion of more than 2/3 but less than the full thickness of the cervical epithelium, and the full thickness of the epithelial layer, but without signs of invasion into the stroma, respectively [66,67]. This distinction was made in the model as reflective of the manner in which data were reported in prior natural history studies of CIN 3 disease, however it is also acknowledged that in clinical practice these classifications may be used interchangeably as distinctions can be subtle.

A study by Westergaard et al. analyzed data for 49 women with biopsy confirmed CIN 3 (severe dysplasia) [68]. Women were followed up with colposcopically directed biopsies and cervical smears at 3 and 9 months post-baseline, with repeat cervical biopsies at least once per year thereafter, if persistent disease was observed. Women progressing to CIS were treated with conization, however, none of the women with persistent severe dysplasia were treated within 12 months. Over a 12 month time period, 47% of women with severe dysplasia progressed to CIS (n = 22) or microinvasive carcinoma (n = 1). The Kataja study, described previously, reported a 12 month risk of progression for severe dysplasia (n = 29) of ~38% [49]. Averaging across the two studies yields a 12 month risk of progression of 43%.

Progression over time was reported through 21 months follow-up in the Westergaard study, with 1 woman treated for severe dysplasia during that period. However, as more women were treated following this time point (with median total follow-up time of 40 months) further data over time were not reported. Using data through 21 months from the Westergaard study, and 45 months from the Kataja study, yields an annual proportion progressing to CIS of 38% and 40% respectively, similar to the 12 month figures. Limitations of these analyses are similar to those described for progression from CIN 2 to CIN 3 (severe dysplasia).

##### HPV 16/18-positive CIN 3 (CIS) to Localized Cervical Cancer

The literature review did not reveal a study meeting the pre-defined eligibility criteria documenting the 12 month progression of untreated CIS to localized invasive cervical cancer (LCC) [International Federation of Gynecology and Obstetrics (FIGO) Stage I]. Based on the limited data available, and calibration to population data for cervical cancer incidence, other cost-effectiveness models of HPV vaccination have assumed a very low rate of annual progression from CIN 2/3 to invasive cervical cancer [7-9]. For instance, Goldie et al. reported a 20-fold range dependent upon a woman's age, varying from as low as 0.2%, to up to 4.0% among women over age 65 [7], while Kulasingam et al. and Sanders et al. have referenced annual transition probabilities of 3–5% with no age-dependency of progression rates [8,9,12].

As noted previously, prior cost-effectiveness analyses have modeled the progression of CIN 2/3 to LCC, rather than CIS to LCC and the existing literature is insufficient for precisely determining the risk of progression to LCC from either state. In the absence of literature-based data, we therefore undertook a strategy of calibrating the model to U.S. cervical cancer rates observed with [47,69], and without [70] cytologic screening. This led to the selection of an overall average 12-month risk of progression from CIS to LCC of 4.1%, represented by two linked compartments (labeled CIS1 and CIS2) with progression rates of 5% and 18% respectively. The low overall annual risk of progression is consistent with the long interval observed between the peak age of HPV infection [71] and cervical cancer detection [70], estimated to be 2–3 decades in duration on average [72].

**Figure 3:**
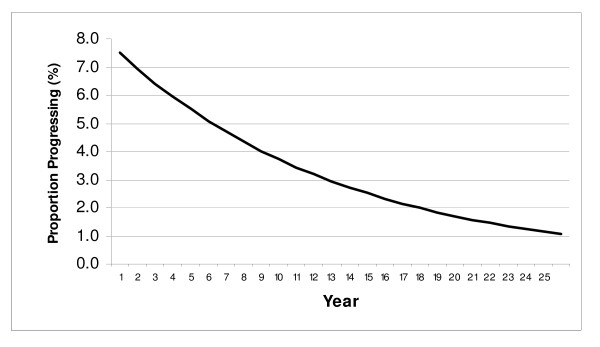
**Annual Proportion of An Incident Cohort Progressing Under An Exponential Distribution**. In this example, an incident cohort at year 0 progresses to a subsequent health state at an annual rate of 0.078, corresponding to a constant annual risk of 7.5%. This results in 7.5% (1*.075) of the original cohort progressing in year 1. By the start of year 10, 49.6% of the original cohort remains in the initial health state, and only 3.7% (.496*.075) progress during year 10. Regardless of the value for risk chosen, the absolute proportion progressing will be highest during year 1 and decline steadily with time. For simplicity, mortality and disease regression are not modeled here.

The decision to divide the CIS state into two compartments was motivated by a comparison of model output with observed data on cervical cancer incidence [69,70]. The model utilizes an exponential distribution to model progression (and regression) which, like Markov processes featured in other cost-effectiveness models [7-9] is characterized by a constant transition rate over time. As seen in the example in Figure 3, with a constant progression rate, the largest proportion of individuals newly entering a given health state will progress to a subsequent health state during the first year, due to a declining denominator in the initial health state during each subsequent year.

**Figure 4:**
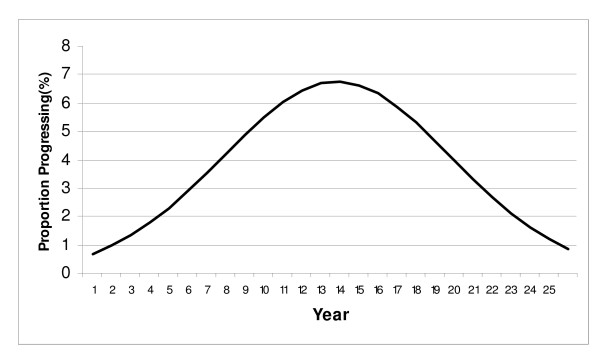
**Illustration of Normally Distributed Progression Over Time In An Incident Cohort**. In this example, an identical average annual rate (0.078) and risk (7.5%) of progression over a 25 year period has been modeled as in figure 3. However, through a transformation, the absolute risk of progression over time has now been rendered normally distributed (with standard deviation of 6) with the largest proportion of individuals now progressing near year 13 rather than year 1.

For health states of shorter duration, with relatively high rates of progression and/or regression, such as for HPV infection and CIN (up to CIS), this formulation may reasonably describe natural history, as time in the health state will be relatively brief regardless of the distributional shape applied to the observed rates. However, for health states of relatively long duration, one may generate results in which individuals transition to another health state much more quickly than is consistent with the natural history of disease. Figure 4 illustrates an identical *average *risk of progression over a 25 year period as that depicted in Figure 3, however, the distribution has now been rendered more normally distributed, with the peak proportion of the initial cohort now progressing during year 10 (7.5%).

**Figure 5:**
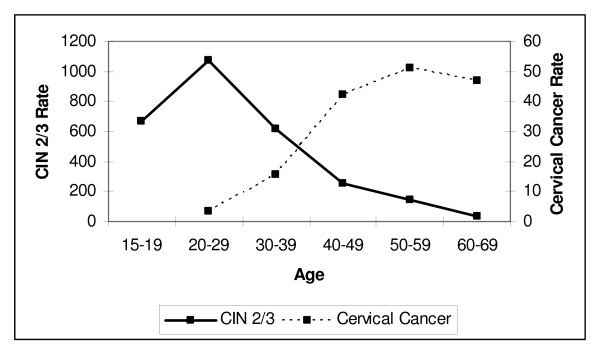
**Incidence of CIN 2/3 Detected Through Screening and Cervical Cancer Incidence Prior to Screening**. Rates are per 100,000 women undergoing routine cytologic screening for CIN 2/3, and per 100,000 women for cervical cancer. The peak incidence of invasive cervical cancer is observed approximately 25–30 years later than for CIN 2/3. **Sources: **CIN 2/3 incidence among screened women (Kaiser Permanente Northwest Health Plan, Portland, Oregon, 1998–2002) [73], Cervical cancer incidence among unscreened women (Connecticut, 1940–1944) [73].

To gain insight into whether a distribution more similar to Figure 3 or to Figure 4 would be most appropriate for modeling the progression of CIS to invasive cervical cancer, we compared age-specific rates of CIN 2/3 to those observed for cervical cancer. From recent population data, the peak incidence of CIN 2/3 has been observed among screened women age 25–29, with a steep decline in incidence thereafter [73,74]. However, data for cervical cancer incidence in Connecticut prior to screening shows a peak incidence among women age 50–59 during the 5-year period from 1940–44 [75]. These age trends are illustrated using data from Insinga et al. [73] and Laskey et al. [75], respectively, in Figure 5.

If one were to utilize a distribution with a constant risk of progression, as depicted in Figure 3, then with the majority of CIN 2/3 cases observed by the late 30 s, if not at an earlier age, one would expect the peak incidence of cervical cancer to be observed around that time. Instead, however, the peak age of cervical cancer incidence in the absence of screening was observed to be in the 50 s in Connecticut [75], as well as in a separate study of cancer incidence among women living in 10 U.S. cities conducted in 1947 [76,77], suggesting that the largest proportions of women progressing to cervical cancer are likely doing so some years after the women have initially entered the CIS state. This is more characteristic of Figure 4, in which a relatively smaller proportion of women progress very quickly to cervical cancer, (as would be disproportionately observed among women diagnosed with cervical cancer following the introduction of screening), with a relatively larger fraction of women progressing to cervical cancer after a number of years (whose cancers would be much more likely to be detected and prevented in the pre-invasive phase with screening).

The division of the CIS state into two compartments was designed to approximate this latter distributional shape, with the relatively slower annual risk of progression from CIS1 to CIS2 (0.05), followed by a faster risk of progression from CIS2 to LCC (0.18), allowing for a delay in the time point of peak progression of CIN 3 to LCC (by 8–10 years) so as to better fit cervical cancer natural history data [70]. Under these assumptions, in the absence of screening and hysterectomy, the model estimates a peak cervical cancer incidence due to HPV 16/18 infection of 43.6 per 100,000 among women age 50–59. This compares to peak rates of cervical cancer incidence, regardless of HPV type, of 50–90 per 100,000 among women age 50–59, observed in Connecticut and 10 U.S. cities prior to the introduction of screening [75]. The model incidence falls near the lower end of this range because approximately 70% of all U.S. cervical cancers are caused by HPV 16/18 infection [16].

##### HPV 16/18-positive LCC to Regional and Distant Cervical Cancer

As was the case for CIS, no studies providing natural history data on progression across cervical cancer stages were found that met the review eligibility criteria. For LCC to regional cervical cancer (RCC) [FIGO Stages II-III], we modeled an annual progression risk of 10%, and for RCC to distant cervical cancer (DCC) [FIGO Stage IV] a risk of 30%, within the ranges assumed in prior HPV natural history models [7,9,22,23]. Progression rates within cervical cancer states were modeled within a single compartment, as they were estimated to be of significantly shorter duration than time spent with CIN3-CIS [23].

#### Progression of HPV 6/11 infection to Histologically Detectable CIN and Anogenital Warts

##### HPV 6/11 infection to CIN 1

The Insinga study described previously was the only analysis meeting review eligibility criteria found to describe the annual risk of clinically diagnosed CIN of any grade following HPV 6/11 infection [43]. The Hoyer study reported the risk of CIN among women with H-R HPV infection [53], however, data were not found in that or other studies for women with L-R HPV infection.

In the Insinga analysis, from among 116 women with incident HPV 6/11 infections, 8.5% were diagnosed with biopsy-confirmed CIN 1 testing PCR-positive for HPV 6/11 infection within 12 months [43]. Through a mean follow-up of 20.6 months, 12.3% of women progressed to CIN 1, for an annualized risk of 7.4%.

##### HPV 6/11 infection to CIN 2/3

In the Insinga study, two woman with incident HPV 6/11 infections were observed to have a CIN 2 lesion testing PCR positive for HPV 6/11 infection within 12 months for a progression risk of 1.9% [43]. No additional progression was observed through 36 months of follow-up. No HPV 6/11 positive CIN 3 cases were observed through 36 months of total follow-up. A review of U.S. studies performing HPV typing of biopsy-confirmed CIN 3 lesions did not reveal any cases testing positive for HPV 6 or HPV 11 infection [43,78,79] and these types have not been observed more frequently than among control women in cervical cancer biopsy specimens [64]. We therefore chose to assume that HPV 6/11 infection did not progress beyond CIN 2.

##### HPV 6/11-positive CIN 1 to CIN 2

In the F.U.T.U.R.E. study analyses described previously, there were 25 incident cases of CIN 1 testing PCR positive for HPV 6/11, none of which progressed to CIN 2 during the course of follow-up (R. Insinga, unpublished data). The model transition probability for this parameter was therefore set to zero.

##### HPV 6/11 infection to Anogenital Warts

In the Winer study described previously, anogenital warts were detected within 12 months in ~57% of women with incident HPV 6/11 infection [41]. Through the mean follow-up time of 38.8 months, anogenital warts were observed in 66.2% of women with incident HPV 6/11 infection, which would translate to an annual risk of progression of 28.5%. However, no new warts were observed among women with incident HPV 6/11 infection during the final 12 months prior to the mean follow-up time, and the 12 month progression figure may therefore be more reliable if the totality of progression occurs soon after infection. The Winer study did not show progression of HPV 16/18 infection to genital warts within 12 months time and we assumed that these types did not progress to warts [41].

#### Duration of HPV 6/11 and HPV 16/18 infections in the Absence of CIN

##### Duration of HPV 16/18 Infections

The duration of HPV infection, in the model, refers to the persistence of type-specific HPV infection up to the point of progression to clinically detectable CIN, or clearance of HPV infection. Only two studies were identified reporting the mean duration of incident HPV 16/18 infections [42,80]. The first, by Richardson et al., censored women at the time of colposcopy, rather than truncating infection duration upon the detection of CIN, and followed women for less than 2 years post-infection, which likely underestimated mean duration due to the censoring of a significant fraction (~50%) of persistent HPV infections at the conclusion of follow-up [80]. The second, by Insinga et al., truncated infection duration at the time of CIN development and followed women for up to 3 years post-infection, with 2.5% of infections persistent in the absence of CIN beyond that time point [42]. The latter study estimated a mean duration of HPV 16/18 infection of 1.2 years and this figure was selected for the model.

##### Duration of HPV 6/11 Infections

From the Insinga study described previously [42], the mean duration of incident HPV 6/11 infections was estimated to be 0.7 years.

#### Existence and duration of acquired immunity following HPV infection

Nearly all previous HPV natural history models used in policy analyses have assumed that women infected with HPV have no protection against subsequent re-infection [7-9,22,24,26]. Data on the existence of elevated antibodies following type-specific HPV infection have been reported [81], however, the potential degree and duration of subsequent acquired immunity to HPV infection are unknown. Several recent clinical trials of HPV vaccines have demonstrated that antibody responses mounted to HPV virus-like particles can provide protection against HPV infection for a period of at least several years [19,20,22]. It would thus seem plausible that natural infection with HPV may also confer some degree of immunity, although available data are insufficient for precise estimation [83,84]. We therefore chose to vary the duration of assumed acquired immunity following HPV infection from 10 years to lifetime in the model.

#### Regression of HPV 16/18 positive CIN

##### HPV 16/18-positive CIN 1 to Negative/HPV 16/18

The regression of CIN 1 lesions testing PCR positive for HPV 16/18 infection was examined in a study of placebo arm enrollees from the F.U.T.U.R.E. I and II trials as described previously. Women with incident CIN 1 lesions were presumed to have regressed if they had at least 2 consecutive negative cervical cytology or histology specimens following diagnosis. Within 12 months, 45.7% of incident HPV 16/18 positive CIN 1 cases (n = 64) were estimated to have regressed.

A study by Sastre-Garau et al. reported that ~18% of prevalent CIN 1 lesions testing positive for HPV 16/18 infection (n = 28) regressed within 12 months [40]. The interval between follow-up visits was generally longer than in the F.U.T.U.R.E. trials, with women observed to have biopsy-confirmed CIN 1 at baseline undergoing a repeat colposcopy and biopsy at 10–12 months and again at approximately 2 years. Regression was defined to occur if CIN was not observed on follow-up colposcopy and biopsy. Averaging results across the two studies yielded an average 12 month risk of regression of HPV 16/18 positive CIN 1 of 32.9%.

Data beyond 12 months were not available from the trial analyses, but over a median follow-up of 24 months, the annual risk of regression in the Sastre-Garau study was approximately 19%. A limitation of both analyses is that cervical HPV typing results were not available during the follow-up periods, with the exception of instances in which histologic specimens were obtained. Thus it is possible that some women with evidence of persistent abnormal cytology/histology harbored disease due to other HPV types following the regression of their HPV 16/18 positive CIN 1 lesions, which may render the reported estimates of disease regression somewhat conservative. Several additional studies meeting review eligibility criteria estimated the risk of regression of CIN 1 without regard to HPV type [48,49,57,85]. The literature review did not yield data on the proportion of women with regressed CIN who remain positive for HPV infection following the disappearance of clinically detectable CIN. A figure of 50% was therefore used as a base case assumption, with variation in sensitivity analyses.

##### HPV 16/18-positive CIN 2 to Negative/HPV16/18

As no studies were identified reporting data for the regression of HPV 16/18-positive CIN 2 lesions, we utilized data from three studies reporting data across all biopsy-confirmed CIN 2 cases. The first two studies, by Kataja and De Aloysio, have been described previously and reported 12 month risks of regression of CIN 2 to HPV/normal of ~2% and 40% respectively [49,65]. In the Kataja study, evidence of regression required a negative result on Pap smear, colposcopy and biopsy at a given visit, while in the De Aloysio study regression was based on the absence of CIN on biopsy only. A third study by Matsumoto et al. reported that ~21% of CIN 2 lesions (n = 36) regressed within 12 months [85]. In the Matsumoto study, women with biopsy confirmed CIN 2 at baseline were followed at 3–6 month intervals with cytology and colposcopy. Regression was based on negative colposcopy and at least 2 consecutive negative Pap smears. Averaging data across the three studies produced a 12-month risk of regression of CIN 2 to normal/HPV of 21%.

The mean or median follow-up time was not reported in the Matsumoto study, however, the mean annual risk of regression through the mean follow-up times in the Kataja and De Aloysio studies were 15% and 23% respectively [49,65]. Limitations of the studies are similar to those described for studies reporting data on the regression of CIN 1.

##### HPV 16/18 positive CIN 2 to CIN 1

Of the three studies just described, only that of De Aloysio also reported on the regression of CIN 2 to CIN 1 over time. In addition to the group regressing to normal/HPV, another 13.3% of women with CIN 2 in that study regressed to CIN 1 within 12 months [65]. This figure was 11% through the mean follow-up time.

##### HPV 16/18 positive CIN 3 (severe dysplasia) to Negative/HPV 16/18

The only study meeting the review eligibility criteria for the regression of CIN 3 (severe dysplasia) was that of Kataja [49]. In that analysis, the 12 month risk of regression of severe dysplasia to normal/HPV was ~11%. Through the mean follow-up time the figure was ~10%.

##### HPV 16/18 positive CIN 3 (severe dysplasia) to CIN 2 or CIN1

The Kataja study did not report data for the regression of severe dysplasia to CIN 2 or CIN 1. However, as was the case for CIN 2, in practice such regression is likely to occur [65]. In the absence of available data, we assumed that the ratio of CIN 3 regressing to Negative/HPV 16/18 versus lower grade CIN was similar to that observed for CIN 2 (1.6:1). This resulted in an estimated regression of CIN 3 to CIN 2 and CIN 1 over 12 months of ~6%, which was divided evenly into 3% to CIN 2 and 3% to CIN 1. Disease progressing beyond severe dysplasia in the model was assumed not to regress.

#### Regression of HPV 6/11 positive CIN and anogenital warts

##### HPV 6/11-positive CIN 1 to Negative/HPV 6/11

The study utilizing F.U.T.U.R.E. I and II trial data was the only analysis identified examining the regression of CIN 1 testing positive for HPV 6/11. The 12-month risk of regression was 55.2% (R. Insinga, unpulished data).

##### HPV 6/11-positive Anogenital Warts to Negative/HPV 6/11

No studies were found documenting the 12 month risk of regression of anogenital warts in the absence of treatment. Although a number of clinical trials have reported wart clearance rates for patients receiving placebo, most have featured follow-up of 3 months or less, and included high proportions of patients with extensive, recalcitrant and previously treated warts [86,87]. Only one study, by Friedman-Kien, was found to report longer-term data for previously untreated genital wart patients [88]. In that analysis, 3 of 8 (37.5%) previously untreated genital wart patients were free of their warts at approximately 20 weeks follow-up. Annualizing results from this small sample yields a 12-month clearance probability of 71%.

However, in practice, individuals with the largest and most extensive warts may seek immediate treatment, and natural clearance rates may be even higher among those patients not seeking physician care. Considering those patients who are treated, the Winer study described previously, reported that 75% of women with incident warts undergoing treatment cleared the warts within 8.0 months [41]. Data through 12 months of follow-up were not reported. However, assuming a constant proportional hazard, the Winer results would convert to a 12 month proportion regressing of 87.5%. Other recent data on incident anogenital wart clearance with a similar length of follow-up in females or in males were not found. In the absence of more suitable data, a 12 month probability of clearance of 87.5% was estimated both for those warts that are treated and those for which treatment is not sought. A population-based study of wart patients in the United Kingdom noted a similar proportion of male and female patients receiving treatment had cleared their warts within 3 months [89]. We therefore assumed that anogenital wart clearance occurred at a similar rate in males and females.

#### Cervical Cancer Mortality (As a % of women within each cervical cancer stage and age grouping)

No studies were found estimating age- and stage-specific annual mortality rates among a representative sample of unscreened women with cervical cancer, as any such analysis would be fraught with ethical and methodological difficulties. As an approximation, data from the Surveillance Epidemiology and End Results Program (SEER) for 1997–2002 were used to estimate the 12 month excess risk of mortality due to cervical cancer, by age group and stage [69]. Excess risk of mortality due to cervical cancer is estimated by SEER through a comparison of age-specific mortality among women with cervical cancer to that observed among women in the same age groups in the general population [90]. Because cervical cancer mortality is highest during the first 12 months following diagnosis [90] use of the 12 month risks allows for the estimation of an average mortality rate in the absence of treatment that is higher than that estimated with treatment, as will be described in a subsequent section. Other data sources for cervical cancer mortality have been used in adaptations of our model to country settings of Taiwan [91] and Mexico [92].

### Clinical Diagnosis and Treatment

Table 4 summarizes clinical diagnostic and treatment variables used in the model. As noted in the Methods, certain clinical parameters (i.e., rates of Pap screening/coverage, rates of hysterectomy) would be expected to vary by country. As in prior reviews of HPV natural history and clinical impact for infectious disease modeling [22,26], it would impractical to attempt to describe these parameters for every country in the world here. Consistent with our recently developed model [14], parameters most likely to vary by country are described for the U.S. population, however data source selection issues may also be applicable to other settings. We also reference data sources used in adaptations of our model to selected other country settings. Unless otherwise noted, type-specific HPV disease data were unavailable for clinical diagnostic and treatment variables.

**Table 4:** Clinical diagnosis and treatment parameters

Parameter	Parameter estimate
Hysterectomy for non-HPV-related conditions, % per year [93]	
15–24 years	0.02
25–29 years	0.26
30–34 years	0.53
35–39 years	0.89
40–44 years	1.17
45–54 years	0.99
≥ 55 years	0.36
Cervical cytology screening, % per year (excluding those with hysterectomy) [47]	
10–14 years	0.6 (0.6)
15–19 years	21.0 (21.0)
20–24 years	44.6 (44.8)
25–29 years	60.4 (61.6)
30–34 years	52.4 (54.9)
35–39 years	46.0 (50.5)
40–44 years	41.0 (48.1)
45–49 years	39.1 (49.1)
50–54 years	38.0 (51.1)
55–59 years	33.2 (46.7)
60–64 years	29.4 (42.5)
65–69 years	26.2 (38.9)
70–74 years	19.4 (29.6)
75–79 years	12.9 (20.1)
80–84 years	7.0 (11.1)
85+	3.4 (5.5)
Women never screened, %	5.0
Liquid-based cytology sensitivity, %	
for CIN 1 [114]	28
for ≥ CIN 2/3 [115]	59
Liquid-based cytology specificity, % [114,115]	94
Colposcopy sensitivity, % [117]	96
Colposcopy specificity, % [117]	48
Symptom development, % per year	
for LCC	4
for RCC	18
for DCC	90
Eradication with treatment, %	
for CIN1 [118-124]	97
for CIN2 [118,122,124,125]	93
for CIN3 [118,122,124,125]	93
for LCC [89]	92
for RCC [89]	55
for DCC [89]	17
for anogenital warts [41]	87.5/year
Persistence of HPV following eradication of CIN, % [127]	34
Persistence of HPV following eradication of cervical cancer, % [129]	47
Persistence of HPV following eradication of genital warts	34
Anogenital wart patients seeking physician care, % [71]	75

CIN = cervical intraepithelial neoplasia; DCC = distant cervical cancer; HPV = human papillomavirus; LCC = localized cervical cancer; RCC = regional cervical cancer

#### Hysterectomy For Non-HPV-related Conditions

Age-specific hysterectomy rates were modeled based on 1994–1999 data reported from the U.S. National Hospital Discharge Survey [93]. Other data sources for hysterectomy rates have been used in forthcoming adaptations of our model to country settings of the United Kingdom [94], Taiwan [95] and Mexico (Unpublished data. Instituto Mexicano de Seguridad Social, D.F., Mexico, 2004).

#### Utilization of Cytology Screening

Nationally representative data on cervical cancer screening are based on self-reported behavior, which has been shown to over-estimate the actual receipt and recency of cervical cancer screening [96-101]. Two large U.S. studies utilizing automated cervical cytology [47] and medical claims [102] databases revealed annual proportions of privately insured women receiving cervical cancer screening that were *lower *than those estimated from nationally representative self-reported data for all women (privately insured, publicly insured and uninsured) [104]. We therefore chose to estimate Pap screening utilization among screened women using validated cytology data from the Kaiser Permanente Northwest (KPNW) health plan [47] and to estimate the proportion of women who are never screened during their lifetimes from data sources that include the uninsured and publicly insured. The decision to separately specify the proportion of women never screened was based on the observation that women with little or no screening to account for a disproportionate share (50–60%) of new cervical cancer cases [104,105].

The KPNW data were selected because they were derived from a large sample of women (n = 150,052), provided a detailed breakdown of annual Pap screening utilization by age group, and distinguished between cervical and vaginal cytology and routine and non-routine Pap testing[47]. The overall proportion of women receiving Pap screening within KPNW each year was observed to be similar to that in a large U.S. claims-based study of women from across the U.S [102,106]. For modeling purposes, two adjustments were made to annual age-specific routine cervical cancer screening rates reported for KPNW. First, the model assumes that women who have undergone removal of the cervix through hysterectomy no longer receive cervical cancer screening. The KPNW cervical cancer screening rates were based on cervical cytologic utilization, and excluded vaginal cytologic screening among women having undergone hysterectomy. However, KPNW screening rates were reported across all women including both those with and without a cervix. We therefore adjusted the rates to reflect screening frequencies among women *with *a cervix using data reported in that analysis on the proportion of women in each age group estimated to have an intact cervix [47].

Second, KPNW screening rates reflect the experiences of both screened (at least once in lifetime) and never screened women. Because we wished to model the never screened population separately, we elected to adjust the rates in the screened population to reflect the removal of never screened women from the denominator. Data on the proportion of women who have never undergone cervical cancer screening in the U.S. are only available from studies featuring self-reported data. As discussed previously, for a variety of reasons [107], the proportion of women who have never been screened in these studies is likely to be under-reported. We chose to model 5% of women as never receiving Pap screening, as this was consistent with the upper end of estimates from the self-reported literature [108,109] and yielded an overall fraction of cervical cancer cases occurring among never screened women (22%) roughly similar to that reported in population-based studies [104].

Other data sources for cytology screening rates have been used in forthcoming adaptations of our model to country settings of the United Kingdom [110], Taiwan [111] and Mexico [112].

#### Liquid-Based Cytology Sensitivity

As most cervical screening in the U.S. is now conducted using liquid-based cytology (LBC) [113] we chose to model test characteristics for that screening method. In selecting studies for analysis of LBC sensitivity, we excluded analyses which did not perform cervical biopsy on at least a random sample of women with normal cytology and colposcopy results. This criterion was established because studies which do not biopsy these women will tend to over-estimate the sensitivity of LBC when CIN or cancer are present in women with normal cytology/colposcopy.

For assessing the sensitivity of LBC for CIN 1, the only study observed to meet this criterion was conducted by Bigras et al. in a non-high-risk Swiss population (n = 13,842) [117]. In the Bigras study, all women with an abnormal Pap smear or high-risk HPV test were referred for biopsy, with a random sample of 502 women with normal Pap and HPV tests also undergoing biopsy. To calculate LBC test characteristics, an extrapolation was made from these two groups of patients to the full sample of women. The sensitivity of LBC for CIN 1 lesions was estimated to be 28%.

The sensitivity of LBC for CIN 2 and CIN 3 were also estimated based on data from the Bigras study. Using data reported in their paper, a sensitivity of 61% was computed for CIN 2 and 55% for CIN 3, with an average sensitivity for CIN 2/3 combined of 59% [115]. In a second study that also selected women with normal cytology and HPV results at random for biopsy, Kulasingam et al. estimated a similar LBC sensitivity for CIN 3 or more severe disease of 57% [12]. Based on these figures we elected to use an LBC Pap test sensitivity for CIN 2/3 or more severe disease of 59% in our base case analysis.

#### Liquid-Based Cytology Specificity

For assessing the specificity of LBC, we relaxed the criteria for study eligibility to include studies in which all women were referred for colposcopy, with a biopsy performed if an abnormality was suspected. Studies conducted exclusively or primarily among women with abnormal cytology results, or for which biopsy results for all grades of CIN (1–3) were not reported were excluded. The decision not to require that biopsy results be reported (or extrapolated) for all women, including those with negative cytology and HPV testing results, was based on the observation from the Bigras analysis that test specificity (98% using either criteria) is much less influenced by this requirement than overall sensitivity for CIN 1–3 (31% vs. 45%) [114]. With a threshold for disease of biopsy-confirmed CIN 1, a specificity of 98% was computed from the Bigras analysis [114]. A specificity of 90% was computed from a second study by Coste et al., conducted among French women undergoing routine cervical cancer screening [115]. In the Coste analysis, all women were referred for colposcopy, with a biopsy performed if an abnormality was suspected [115,116]. Based on these analyses, we estimated an average specificity for LBC with a disease threshold of CIN 1 of 94%.

#### Colposcopy Sensitivity

A meta-analysis by Mitchell et al. examined studies in which women with an abnormal Pap smear underwent both colposcopy and colposcopically directed biopsy, with results tabulated by grade of CIN and cancer [117]. Nine studies met the review eligibility criteria, with a mean sensitivity for colposcopy to detect histologic abnormalities (including CIN 1–3 and cancer) of 96%.

#### Colposcopy Specificity

The meta-analysis by Mitchell et al. also reported a mean specificity for colposcopy of 48%, and this figure was used in the model [117].

#### Symptom Development Among Cervical Cancer Patients

It was assumed that women not diagnosed with asymptomatic cervical cancer through screening would be diagnosed if symptoms developed. The literature review did not identify any studies describing stage-specific symptom development in a representative cross-section of patients with cervical cancer. A difficulty in interpreting existing case series is that it is not possible to determine the true number of women within the population who harbor asymptomatic cervical cancer. In the absence of such data, we reviewed assumptions used in prior cost-effectiveness analyses [7,9,21,22] and chose an annual probability of initially developing symptoms for LCC of 4%, RCC of 18%, and DCC of 90%.

#### Eradication of CIN following treatment

For estimating the rate of eradication of CIN following treatment, we selected studies evaluating the performance of the commonly used LEEP procedure, subject to the following criteria: 1) Stratified reporting by CIN grade of the number of subjects with confirmed pre-treatment histology of CIN 1, 2 or 3; 2) Post-treatment follow-up of all women within 12 months with colposcopy and/or biopsy; 3) Definition of recurrent or residual disease as CIN 1 or more severe histology; 4) Stratified reporting of the number of subjects with recurrent/residual disease according to the grade of pre-treatment histology.

Seven studies evaluating the treatment of CIN 1 met the review eligibility criteria, comprising 417 cases [118-124]. A total of 14 cases of residual/recurrent CIN were reported in these studies, for a treatment eradication rate of 97%. For CIN 2, seven eligible studies (n = 403, 30 cases of residual/recurrent CIN) [118-124] yielded a treatment eradication rate of 93%, and for CIN 3, eight eligible studies (n = 1,565, 109 cases of residual/recurrent CIN) [118-122,124-126] a rate of 93%. Limitations of the pooled analysis included that data were insufficient to evaluate treatment efficacy for HPV 16/18 or 6/11 lesions specifically and, with the exception of one small study [118] women with negative colposcopies during follow-up were not biopsied.

#### Eradication of cervical cancer following treatment

For cervical cancer, it was assumed that women surviving for more than 5 years following diagnosis were cured of disease [89]. We therefore utilized 5-year relative survival rates by stage from the SEER program to estimate the proportion of women whose cancers were successfully eradicated with treatment [90]. For the period 1995–2001, the 5-year relative survival rate for localized cervical cancer in the SEER program was 92%, for regional cancer 55%, and for distant cancer 17%. An age and stage-specific mortality rate was modeled for women not cured of their cancers, as described in the previous section on cervical cancer mortality.

#### Eradication of anogenital warts following treatment

For anogenital warts, current treatments often require multiple applications and often fail, requiring second and third-line therapies [87]. Rather than modeling treatment algorithms explicitly, a global 12-month clearance rate with treatment of 87.5% was assumed, based on the Winer study as previously described [41].

#### Persistence of cervical HPV infection following eradication of CIN

Women whose CIN is successfully removed and who do not develop recurrent CIN may still harbor HPV infection transmissible to others. To inform this model parameter, we searched for studies in which: 1) HPV testing was reported specifically for HPV types 16/18 or 6/11; 2) HPV DNA testing was performed on pre-treatment biopsies or treatment specimens of confirmed CIN histology; 3) Follow-up for all women and HPV testing occurred within 6 months post-treatment; 4) Colposcopy was performed on all women during follow-up to confirm the eradication of CIN lesions; 5) Persistent CIN disease during follow-up (to be excluded) was confirmed by histology. One previous study was identified which met these criteria. Cruickshank et al. studied women who were treated for CIN 3 via LEEP and laser ablation, had negative Pap smears and colposcopic assessments at 6 months, and remained free of disease thereafter for a minimum of 5 years [127]. HPV testing and typing was conducted using tissue obtained from the diagnostic biopsy and cytologic material from the 6 month follow-up smear. Among women with HPV 16/18 infection detected in the diagnostic biopsy (n = 35), 12 (34%) had detectable HPV 16/18 infection at their 6 month post-treatment smear. We therefore assumed that this percentage of women successfully treated for CIN maintained cervical HPV infection following therapy as other studies with similarly rigorous HPV typing and follow-up regimens were not identified. It should be noted that the clearance of HPV infection in the cervix, coinciding with treatment for CIN, may have little influence with respect to the clearance of HPV infection co-existing at other anogenital sites, although in the model HPV infections were not site-specific [15]. Paraskevaidas et al., have also reviewed the persistence of HPV infection more generally (regardless of HPV infection type), following CIN eradication [128].

#### Persistence of HPV infection following eradication of cervical cancer

One study was identified through the literature review evaluating the PCR-based type-specific persistence of HPV infection following treatment for cervical cancer [129]. Fen et al. performed HPV typing of cervical swabs prior to hysterectomy among women with Stage I and II cervical cancers and compared results to swab samples taken from the residual endocervix or vaginal mucosa 6–24 months following treatment. Among women with HPV 16/18 infection prior to treatment (n = 30), 46.7% had persistent HPV infection with the same type following hysterectomy [129]. Limitations of the study with respect to the present review include that results were not stratified by recurrence status and the time interval to post-treatment HPV testing was at least several months. Other studies overcoming these limitations were not found.

#### Persistence of HPV infection following eradication of genital warts

The literature review identified two studies comparing the type-specific prevalence of HPV 6/11 infection prior to and following successful eradication of genital warts. The first, by Arany et al., evaluated 6 patients successfully treated via interferon therapy for HPV 6/11 positive genital warts and found 100% clearance of HPV 6/11 infection within normal skin at the wart site at 6–12 weeks follow-up [130] The second, by Syed et al., also reported complete elimination of HPV 6/11 infection at 12 months following eradication of warts with imiquimod cream, however, the number of patients testing positive for HPV 6/11 infection at baseline, and negative at follow-up, were not specified [131]. Limitations of both studies for purposes of the present review included a lack of information as to whether HPV infection was present on follow-up across a sampling of anogenital sites. Because the sample size in the first study was quite small, and the time to follow-up PCR testing in the second study rather long, we assumed a similar rate of persistence of HPV infection following eradication of anogenital warts as estimated for CIN (34%) in the model.

#### Anogenital wart patients seeking physician care

Not all patients who harbor anogenital warts will be aware of their presence or seek physician care [132,133]. In the Winer study, used to estimate the rate of anogenital warts following incident HPV 6/11 infection, all women underwent Pap screening, colposcopy and HPV testing at 4 month intervals [41]. As a result of the frequent clinical examinations, it is possible that some women who would have otherwise remained undetected were diagnosed and treated for their warts. Although precise data are unavailable, Chesson et al. estimated that approximately 25% of patients with anogenital warts will not seek or require physician care and we utilized this figure [72].

## Discussion

Natural history models of HPV disease are increasingly being used to inform policy making for existing and emerging technologies [7-11,13-15,21,24,44]. This paper has critically reviewed the evidence base for modeling the natural history, clinical diagnosis and treatment of cervical HPV disease and anogenital warts, available at the time of development of our multi-HPV type model [14]. Prior reviews and meta-analyses of the natural history of HPV disease have typically pooled data from studies of varying quality and design (e.g., not distinguishing between cervical cytology and histology) for the purposes of analysis [27-30]. A unique feature of this review has been the utilization of stricter study eligibility criteria in an attempt to select more homogeneous data sources perceived to be of the highest quality presently available. Given that the quantity of eligible studies was small for a number of model parameters, in conducting policy evaluations, modelers should be prepared to conduct sensitivity analyses varying those parameters found to be most influential to the study outcomes of interest [7-11,13-15,21,24,44]. We should also note that the relatively small number of studies available to inform the modeling parameters was an impetus for us to conduct additional studies to better characterize these inputs, reflecting several of the references discussed in this review [42,43,47,73].

Given the breadth and complexity of HPV disease modeling, we have not comprehensively reviewed data to inform all possible model structures and permutations, or every potential limitation of existing studies to inform such models. Furthermore, although we attempted to be thorough in our review, it is also possible that our database and non-database search strategies may have missed some publications of relevance as we did not search all publicly available scientific literature databases (e.g., EMBASE, PsycLIT). However, the present review has attempted to highlight available data sources and associated major strengths and limitations for informing parameters common to many existing models.

## Conclusion

Knowledge of the natural history of HPV disease has been considerably enhanced over the past two decades, through the publication of an increasing number of relevant studies. However, considerable opportunity remains for advancing our understanding of HPV natural history and the quality of associated models. For instance, more studies examining the type-specific progression and regression of HPV disease are needed, both for more accurate modeling of disease natural history and to inform policymaking for technologies such as vaccines that target specific HPV types [7,14,15]. Also, additional studies stratifying progression and regression rates for CIN by age would be helpful in resolving the issue of whether these rates are constant across age or age-dependent. Finally, studies exploring the potential duration of acquired immunity following HPV infection are needed.

For many disease areas, modeling is an iterative process. As one's understanding of disease natural history and clinical management are advanced, existing models can be adapted to incorporate new evidence. Comprehensive literature reviews for HPV disease and other health conditions can play an important role in assisting modelers and policymakers in critically evaluating the current evidence base and identifying areas for future study.

## Abbreviations

AGC: atypical glandular cells; ALTS: ASCUS/LSIL triage study; ASCUS: atypical squamous cells of undetermined significance; CIN: cervical intraepithelial neoplasia; CIS: carcinomain situ; DCC: distant cervical cancer; FIGO: International Federation of Gynecology and Obstetrics; H-R: high-risk; HPV: human papillomavirus; HSIL: high-grade squamous intra-epithelial lesion; KPNW: Kaiser Permanente Northwest; L-R: low-risk; LBC: liquid-based cytology; LCC: localized invasive cervical cancer; LEEP: loop electrosurgical excision procedure; LSIL: low-grade squamous intra-epithelial lesion; PCR: polymerase chain reaction; RCC: regional cervical cancer; SEER: Surveillance Epidemiology and End Results Program.

## Competing interests

The authors are employed by, and may hold stock in, Merck & Co., Inc., which funded the article-processing charge for this study.

## Authors' contributions

RI conceived of the study and conducted the literature review, data extraction and analysis, and initial drafting of a manuscript. EE and ED assumed a primary role in defining the data parameters to be reviewed and assisted in the drafting of the manuscript and supplementary data analyses. All authors read and approved the final manuscript.

## Pre-publication history

The pre-publication history for this paper can be accessed here:

http://www.biomedcentral.com/1471-2334/9/119/prepub
